# Developing a university-accredited Lean Six Sigma curriculum to overcome system blindness

**DOI:** 10.1093/intqhc/mzz074

**Published:** 2019-12-22

**Authors:** Martin McNamara, SeÁn Paul Teeling

**Affiliations:** 1 UCD School of Nursing, Midwifery and Health Systems, College of Health and Agricultural Sciences, Dublin 4, Ireland; 2 Mater Lean Academy, Mater Misericordiae University Hospital, Eccles St., Dublin 7, Ireland

**Keywords:** Lean Six Sigma, education, curriculum, system blindness

## Abstract

This paper discusses the development of a Lean Six Sigma (LSS) postgraduate education programme that has enabled the delivery of over 90 quality improvement projects led by its graduates across 50 healthcare organizations in Ireland. A key success factor in embedding and sustaining LSS in these organizations was the accreditation by a major, national, research-intensive university of the LSS education programme from which the students graduated. To ensure the programme’s approval by the university it was necessary to contextualize LSS within established conceptual frameworks. This helped counter misconceptions that what was proposed was technical training in tools and techniques to provide quick fixes for routine healthcare process issues. Two related conceptual frameworks were selected to frame the curriculum: Senge’s Fifth Discipline and Deming’s System of Profound Knowledge. This paper focuses on how a central element of both frameworks, systems thinking or appreciation for a system, was enacted in the curriculum using Oshry’s work on system blindness. Showing how systems thinking was conceptualized in the curriculum established the legitimacy and credibility of the programme within academia. This led to the approval of the first university-accredited graduate programme in LSS for healthcare in Ireland.

This paper discusses the development of a postgraduate Lean Six Sigma (LSS) education programme that has enabled the delivery of over 90 quality improvement projects led by its graduates across 50 healthcare organizations in Ireland. A key success factor in embedding and sustaining LSS in these organizations was the accreditation by a major, national, research-intensive university of the LSS education programme from which the students graduated [1–3]. To help gain acceptance and approval within academia as a postgraduate programme we designed a curriculum that situated LSS within conceptual frameworks that address the need to transform leadership and management practices. This was necessary to comply with university accreditation and quality assurance criteria, and to ensure a genuinely transformational student experience. This countered any misconception that what was proposed was technical training in tools, techniques and templates to provide quick fixes to routine healthcare process issues. Two related conceptual frameworks were selected to frame the curriculum: Senge’s Fifth Discipline [4] and Deming’s System of Profound Knowledge [5]. The focus of this paper is on how a central element of both frameworks, systems thinking [4] or appreciation for a system [5], was enacted in the curriculum using Oshry’s work on overcoming system blindness [6, 7].

Mazzocato *et al.* [8] note that a key component of successful implementation of LSS in healthcare is an education programme that enables systems vision. A narrow focus on tools and techniques means that systemic factors such as leadership and management practices are neglected. Antony *et al.* [9] stress that LSS implementation must not be limited to operations but must be employed strategically to ensure sustainability. According to Graban [10]:

Lean is an approach that can support employees and physicians, eliminating roadblocks and allowing them to focus on providing care. Lean helps break down barriers between disconnected departmental ‘silos’, allowing different hospital departments to better work together for the benefit of patients (p. 1)

Designing a university postgraduate education programme that enables students who were trained and often work in such silos to recognize and transcend barriers is essential. To sustain LSS programmes, healthcare organizations must ensure that their management and leadership are educated to foster ‘a strategic climate, which focuses…employees on quality, efficiency and innovation’ ([11] p. 2911). The common thread running through these arguments is appreciation of systems [5]. Framing LSS education programmes within conceptual frameworks that emphasize systems thinking [4] also anchors it to the values on which Lean was founded and avoids reducing LSS to a decontextualized toolkit. These values include harmony, loyalty and consensual decision-making, all springing from the central principle of respect for persons [12].

To ensure that graduates of the university programme developed a deep appreciation of systems [5], we drew on Oshry’s [6, 7] work on overcoming system blindness. Oshry [6] uses this term to refer to a pervasive lack of appreciation of how our experience of ourselves, others and our organizations is shaped by the structure and processes of the systems we find ourselves in. He shows that all organisational systems have predictable conditions that prevail at different levels and positions in the organization. He also discusses predictable traps and high-leverage opportunities to avoid them. System blindness causes us to regard particular patterns of behaviours as personal or situational when they are, in fact, systemic. Education in Lean Six Sigma and other improvement initiatives founders when it fails to enable students to think systemically and to consider the roles that they and others play in maintaining a system’s structure and processes [6, 7].

System blindness has corrosive consequences that permeate organisational life. It limits the potential contribution to the system of employees at all levels in the organization, top, middle and bottom. It also constrains the contributions of external stakeholders, including patients and their families [6, 7]. Oshry draws attention to how those charged with shaping systems are often too burdened by unmanageable complexity to do so. He also shows how those who deliver care can become oppressed by what they perceive to be remote and indifferent managers. Those whose principal role is that of systems integration, middle managers, become too confused and torn between the conflicting demands and priorities of their managers and reports to integrate effectively. Meanwhile, the ultimate system validators, those in receipt of healthcare, regularly feel that healthcare delivery systems are insufficiently responsive to their needs [6, 7].

Oshry describes five types of system blindness: spatial, temporal, relational, process and positional [6, 7]. We suffer from spatial blindness when we see our part of the system but not the whole; what is happening to us but not to others. We suffer from temporal blindness when we don’t see the history of the present, the story of our system that has brought us to this point in time. We suffer from relational blindness when we fail to appreciate that we are always in systemic relationship to one another and that our relative position in an organization structures our relationships. We suffer from process blindness when we fail to recognize the importance of four fundamental organisational processes. These processes are:

- Individuation (a tendency to separate);
- Integration (a tendency to work together);
- Differentiation (a tendency to emphasize difference and distinctiveness), and
- Homogenization (a tendency to emphasize shared characteristics and commonality).

Process blindness results from our failure to appreciate, first, the relative balance among these four processes, second, the relative intensity with which they are expressed in different contexts and, third, the part we play in strengthening and weakening them. Finally, we suffer from positional blindness when we see only fixed positions battling other fixed positions but don’t appreciate the uncertainty and ambiguity underlying those positions, the conditions associated with them and the predictable patterns of behaviour that those conditions evoke.

In summary, we located LSS education and practice within established conceptual frameworks [4, 5]. We clarified how key concepts, such as systems thinking, were to be conceptualized, taught and applied to practice across a range of clinical contexts. This established the legitimacy and credibility of the programme within academia, resulting in the approval of the first university-accredited postgraduate programme in LSS for healthcare in Ireland. The programme’s graduates have gone on to become pioneers in leading and delivering LSS projects contextualized for the Irish healthcare system including:

- improved drug round processes releasing nursing time to care [1]
- redesigning hip fracture pathways [2]
- improving day of surgery admission rates [3]
- releasing pharmacy and nursing staff time by redesigning controlled drug ordering processes [13]

These projects have improved the staff and patient experience of delivering and receiving care, and clinical outcomes [1–3, 13]. The LSS education programme has built upon the self and system awareness that Oshry’s [6, 7] work raises, and encouraged students to locate their LSS improvement projects in their proper systemic contexts. This has enabled them to take account of, and address, the spatial, temporal, relational, process and positional dynamics that so often undermine creativity and innovation, sabotage productive partnerships and arrest change programmes [4–7].

## References

[1] Kieran M, Cleary M, De Brún A et al Supply and demand: application of Lean Six Sigma methods to improve drug round efficiency and release nursing time. Int J Qual Healthc 2017;29:803–9. 10.1093/intqhc/mzx106 29025066

[2] O’Toole R, Murphy C, Hogan K et al 057 Utilisation of Lean Six Sigma process improvement methodologies in acture Hip fracture care of the older person. Age Ageing 2016;45:ii13–ii5.

[3] Brown R, Grehan P, Moore E et al Development of the First Thoracic Enhanced Recovery Programme (TERP) in the Republic of Ireland. Society for Cardiothoracic Surgery (SCTS) 2017.

[4] Senge PM The Fifth Discipline Fieldbook: Strategies and Tools for Building a Learning Organization. London: Brealey, 1994.

[5] Deming WE The New Economics for Industry, Government, Education, 3rd edn Cambridge, Massachusetts: MIT Press, 2018.

[6] Oshry B Seeing Systems: Unlocking the Mysteries of Organisational Life. San Francisco, California: Berrett-Koehler Publishers, 2007.

[7] Oshry B Context, Context, Context: How Our Blindness to Context Cripples Even the Smartest Organizations. Cornwall: Triarchy Press, 2018.

[8] Mazzocato P, Holden RJ, Brommels M et al How does Lean work in emergency care? A case study of a Lean-inspired intervention at the Astrid Lindgren Children’s hospital, Stockholm, Sweden. BMC Health Serv Res 2012;12:28. 22296919 10.1186/1472-6963-12-28 PMC3298466

[9] Antony J, Downey‐Ennis K, Antony F et al Can Six Sigma be the ‘cure’ for our ‘ailing’ NHS? Leadership Health Serv 2007;20:242–53. 20698097

[10] Graban M Lean Hospitals**—**Improving Quality, Patient Safety, and Employee Engagement, 2nd edn Florida: CRC Press, 2012. 10.1177/193758671300700111 24554321

[11] Huijsman R, Paauwe J, de Koeijer RJ ‘Toward a conceptual framework for exploring multilevel relationships between Lean Management and Six Sigma, enabling HRM, strategic climate and outcomes in healthcare. Int J Hum Res Manage 2014;25:2911–25.

[12] Suárez-Barraza MF, Ramis-Pujol J, Kerbache L Thoughts on kaizen and its evolution. Three different perspectives and guiding principles. Int J Lean Six Sigma 2011;2:288–308.

[13] Creed M, McGuirk M, Buckley R et al Using Lean Six Sigma to improve controlled drug processes and release nursing time. J Nurs Care Qual 2019;34:236–41. 30198954 10.1097/NCQ.0000000000000364

